# Jejunal villus absorption and paracellular tight junction permeability are major routes for early intestinal uptake of food-grade TiO_2_ particles: an in vivo and ex vivo study in mice

**DOI:** 10.1186/s12989-020-00357-z

**Published:** 2020-06-11

**Authors:** Christine Coméra, Christel Cartier, Eric Gaultier, Olivier Catrice, Quentin Panouille, Sarah El Hamdi, Kristof Tirez, Inge Nelissen, Vassilia Théodorou, Eric Houdeau

**Affiliations:** 1 Toxalim (Research Centre in Food Toxicology), Toulouse University, INRAE, ENVT, INP, Toulouse, France; 2 LIPM, Université de Toulouse, INRAE, CNRS, Castanet-Tolosan, France; 3 VITO (Flemish Institute for Technological Research), Mol, Belgium

**Keywords:** Titanium dioxide, Food additive, Nanoparticles, Intestinal absorption

## Abstract

**Background:**

Food-grade TiO_2_ (E171 in the EU) is widely used as a coloring agent in foodstuffs, including sweets. Chronic dietary exposure raises concerns for human health due to proinflammatory properties and the ability to induce and promote preneoplastic lesions in the rodent gut. Characterization of intestinal TiO_2_ uptake is essential for assessing the health risk in humans. We studied in vivo the gut absorption kinetics of TiO_2_ in fasted mice orally given a single dose (40 mg/kg) to assess the ability of intestinal apical surfaces to absorb particles when available without entrapment in the bolus. The epithelial translocation pathways were also identified ex vivo using intestinal loops in anesthetized mice.

**Results:**

The absorption of TiO_2_ particles was analyzed in gut tissues by laser-reflective confocal microscopy and ICP-MS at 4 and 8 h following oral administration. A bimodal pattern was detected in the small intestine: TiO_2_ absorption peaked at 4 h in jejunal and ileal villi before returning to basal levels at 8 h, while being undetectable at 4 h but significantly present at 8 h in the jejunal Peyer’s patches (PP). Lower absorption occurred in the colon, while TiO_2_ particles were clearly detectable by confocal microscopy in the blood at 4 and 8 h after treatment. Ex vivo, jejunal loops were exposed to the food additive in the presence and absence of pharmacological inhibitors of paracellular tight junction (TJ) permeability or of transcellular (endocytic) passage. Thirty minutes after E171 addition, TiO_2_ absorption by the jejunal villi was decreased by 66% (*p* < 0.001 vs. control) in the presence of the paracellular permeability blocker triaminopyrimidine; the other inhibitors had no significant effect. Substantial absorption through a goblet cell (GC)-associated pathway, insensitive to TJ blockade, was also detected.

**Conclusions:**

After a single E171 dose in mice, early intestinal uptake of TiO_2_ particles mainly occurred through the villi of the small intestine, which, in contrast to the PP, represent the main absorption surface in the small intestine. A GC-associated passage and passive diffusion through paracellular TJ spaces between enterocytes appeared to be major absorption routes for transepithelial uptake of dietary TiO_2_.

## Background

Titanium dioxide (TiO_2_) is manufactured at high production volumes for various industrial applications, mainly as a pigment (e.g., paints, cosmetics) and for photocatalytic properties [1]. In the food industry, food-grade TiO_2_ (E171 in EU) is mainly used as a whitening and brightening agent added to confectionery (candies, chewing gum) and ultra-processed foods [1–4]. The food additive E171 shows a broad size distribution of particles (diameters of 30 to 400 nm), with 10 to 45% of them in number in the nanosized range (i.e., ≤100 nm) depending on the commercial source [1, 5–7]. The Food and Drug Administration approved this food additive to be supplemented to up to 1% of the weight of the food matrix, while in Europe, the Joint FAO/WHO Expert Committee on Food Additives authorized E171 *ad quantum satis* in foodstuffs, on the basis of the very low absorption of TiO_2_ by the gut. However, despite a low rate of intestinal absorption, estimated between 0.1 and 0.6% of the initial dose in humans and rodents [8–11], recent animal studies have emphasized the oral toxicity of food-grade TiO_2_ after chronic exposure at human relevant levels. Reported hazards include intestinal and systemic immune dysfunction, colonic microinflammation, until the development of spontaneous preneoplastic lesions in the colon (rats), and the promotion of tumor formation in chemically induced colon cancer models (rats and mice) [7, 12]. Recently, food safety authorities recommended additional toxicity testing to establish a health-based guidance value for food-grade TiO_2_ [3]. This requires supplemental studies designed to determine in vivo the absorption sites of TiO_2_ particles from the food additive E171 as it passes through the gut, along with information on the mechanisms of particle uptake. In humans, bimodal passage into the bloodstream has been reported after a single oral dose of food-grade TiO_2_ given to volunteers, with an identifiable absorption starting at 1 h and peaking at 6 h after ingestion [13]. Such long-lasting intestinal absorption with a delayed peak for lumen-to-blood passage led the authors to hypothesize two routes for particle uptake from the gut that remain to be precised in vivo. Previous in vitro studies, using a nanoparticle form of TiO_2_ (i.e., 100% nanosized) showed that the microfold cells (M cells) lining the domes of PP were a major absorption site of TiO_2_ particles, in contrast to Caco-2 cells, which were used as a model for normal enterocytes [14]. In mice orally exposed to nanosized TiO_2_ particles by single gavage, titanium (Ti)-rich regions have been reported both in intestinal villi and in PP 6 h after treatment [14]. However, the kinetics and absorption rates for TiO_2_ particle uptake along the gut remain to be specified, particularly the respective contributions of diffuse PP sites compared to absorptive enterocytes, the latter of which represent most of the surface area of the gut epithelium. Furthermore, from a mechanistic point of view, the relative contributions of transcellular (i.e., endocytosis) and paracellular (i.e., through intercellular spaces between enterocytes) pathways for TiO_2_ translocation remain to be determined. For risk assessment purposes, it is crucial to conduct such experiments using food-grade TiO_2_ matter that displays mixed particle sizes, and in vivo experiments are required to determine the prevalence and location of TiO_2_ particle uptake from the food additive E171 along the intestine. In this study, we conducted experiments in mice that received a single dose of E171 to determine the spatiotemporal absorption pattern of dietary TiO_2_ particles along the intestine and their transfer to the blood. Trans- and paracellular routes for transepithelial TiO_2_ passage were studied ex vivo on isolated intestinal loops in anesthetized mice in the presence and absence of selective pharmacological blockades.

## Results

### Kinetic of TiO_2_ absorption along the intestine and systemic passage

**Fig. 1:**
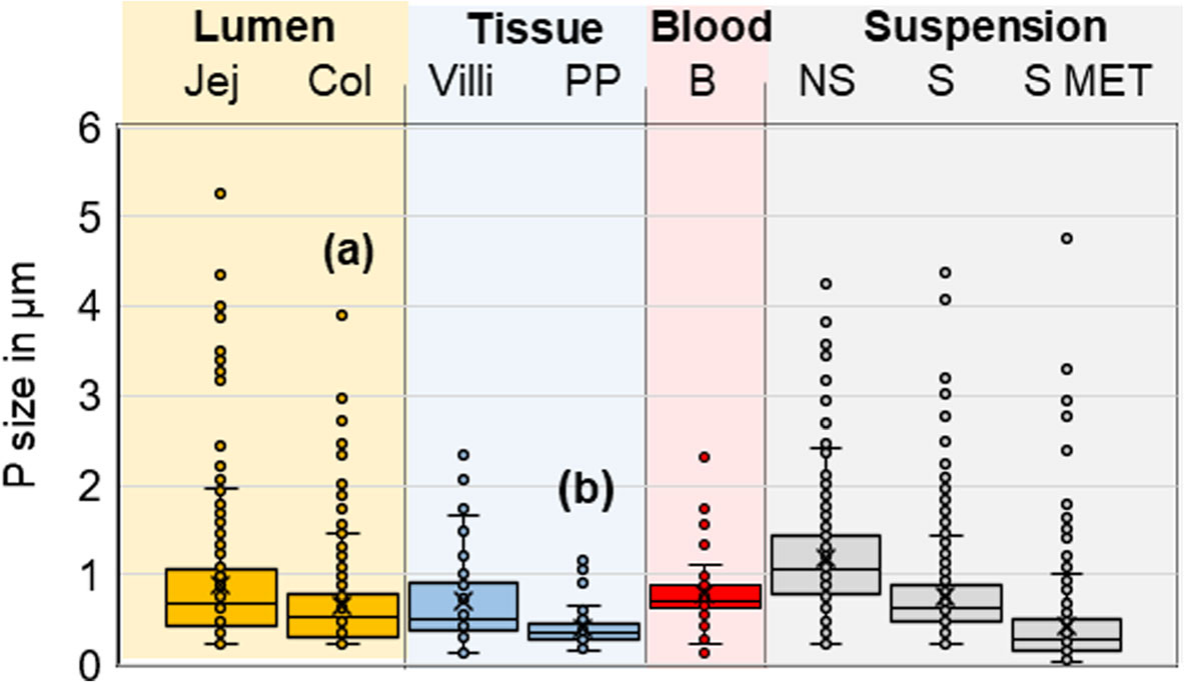
Analysis of TiO_2_ particle sizes. Size measurement of TiO_2_ particles detected in the intestinal lumen of jejunum or colon (lumen Jej and Col), in the jejunal villi and PP (Tissue Villi and PP), in the blood (B) and in the initial suspension (Suspension) before sonication (non-sonicated or NS) or after sonication (S). Confocal size measurement of the TiO_2_ particles was performed at high magnification with dual determination by confocal laser reflexion and by classic bright field observation. In addition, the latter S suspension was analysed by TEM (S MET), for comparison. Statistical significant difference between (a) Lumen Jej and Lumen col. (*p* < 0.001) and (b) in PP compared to B (*p* < 0.05), S and Lumen Jej (*p* < 0.001)

The primary particle size distribution in the E171 batch used herein has been previously characterized by transmission electronic microscopy (TEM), and ranged between 20 and 340 nm in diameter [6, 7]. In the sonicated E171 suspension orally administered to mice, the TiO_2_ particles partly agglomerated in water, thus increasing the overall size of TiO_2_ particles to a range of 33 (isolated particles and nanosized aggregates) to 4750 nm (large agglomerates) as measured by TEM (average size: 485 ± 32 nm, *n* = 354) (Fig. 1). We then used laser-reflecting signals of TiO_2_ measured by confocal microscopy to assess the fraction of TiO_2_ particles present in initial suspension, jejunal or colonic luminal contents, intestinal mucosae and blood. The particles detected by this approach are more or less agglomerated forms appearing as a bright green signal and called TiO_2_ particles throughout this study. Sonicated TiO_2_ suspensions in water were in a larger size range of 220 to 4400 nm (767 ± 37 nm, *n* = 371) compared to TEM, due to lower resolution (Fig. 1). All tissue sections along the gut showed a weak basal level of intrinsic reflection, which was setted as background, permitting easy identification of the much higher reflectance of TiO_2_. In mice receiving a single oral dose of E171 (40 mg/kg), the number of laser-reflecting TiO_2_ particles significantly increased in the lumen of the upper intestine compared to that of control mice (Fig. S2), as previously shown in rats [7]. The particles exhibited similar sizes to those observed in the E171 gavage bolus (Fig. 1), showing no further agglomeration of TiO_2_ particles during their transit. The particle size was even smaller in the colonic (650 ± 25 nm, *n* = 371) compared to the jejunal lumen (871 ± 40 nm, *n* = 332), *p* < 0.001), suggesting a decrease in the agglomeration state of TiO_2_ matter as it moved to the distal intestine (Fig. 1c).

**Fig. 2:**
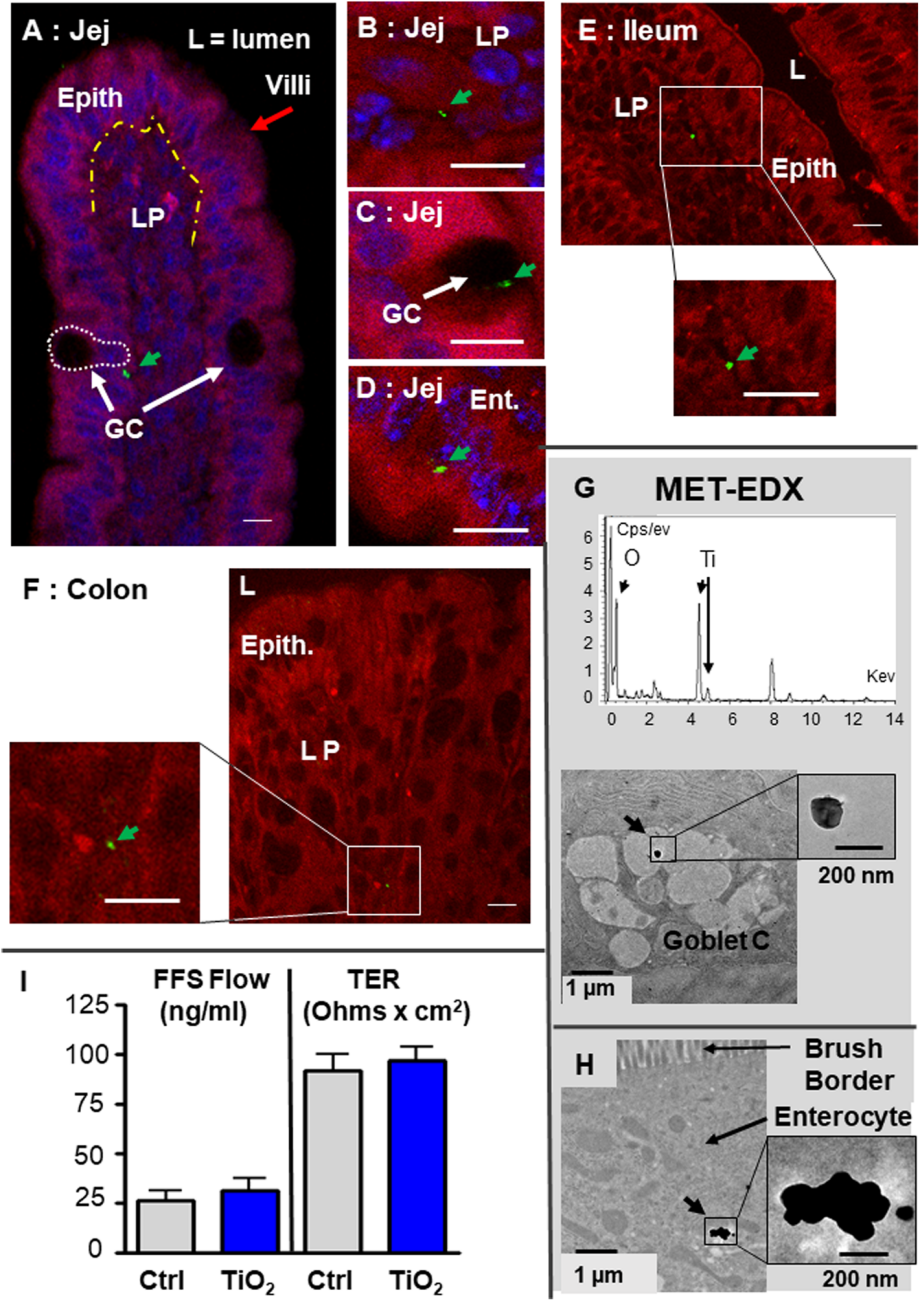
Confocal and TEM localization of TiO_2_ particles in jejunal villi, 4 h after E171 oral administration. **a-f** In the confocal analysis, the TiO_2_ particles are apparent in green (and overlined by green arrows), the tissue auto-fluorescence in red and the nucleus labelled by Dapi in blue for some images **a**-**d**. **a** Absorption in jejunal villus showing its organization, the limit between the epithelial monolayer (Epith) and the lamina propria (LP) is indicated by a yellow dashed line. The GC are indicated by white arrows and one is circled by a white dashed line. A TiO_2_ particle is visible in or near a GC **(a, d)**, in the lamina propria (**b**) or in the epithelium **(d)**. **g, h** TEM associated to EDX analysis: TiO_2_ particles are detected in the mucin-rich vesicles of a GC **(g)** or in the basal side of an enterocyte **(h)**. **i** Intestinal permeability measurement using Ussing chambers in the jejunum from controls (Ctrl) and TiO_2_ treated animals (TiO_2_). The jejunum was recovered 4 h after a single oral dose of E171, longitudinally opened and transferred in a Ussing chamber. The transfer of FFS from the luminal to the serosal side of the jejunum is measured (FFS flow expressed in ng/ml) and the transepithelial resistance of the tissue (TER in Ohms x cm^2^ of tissue). Bars = 10 μm in confocal microscopy, and 1 μm or 200 nm in TEM

**Fig. 3:**
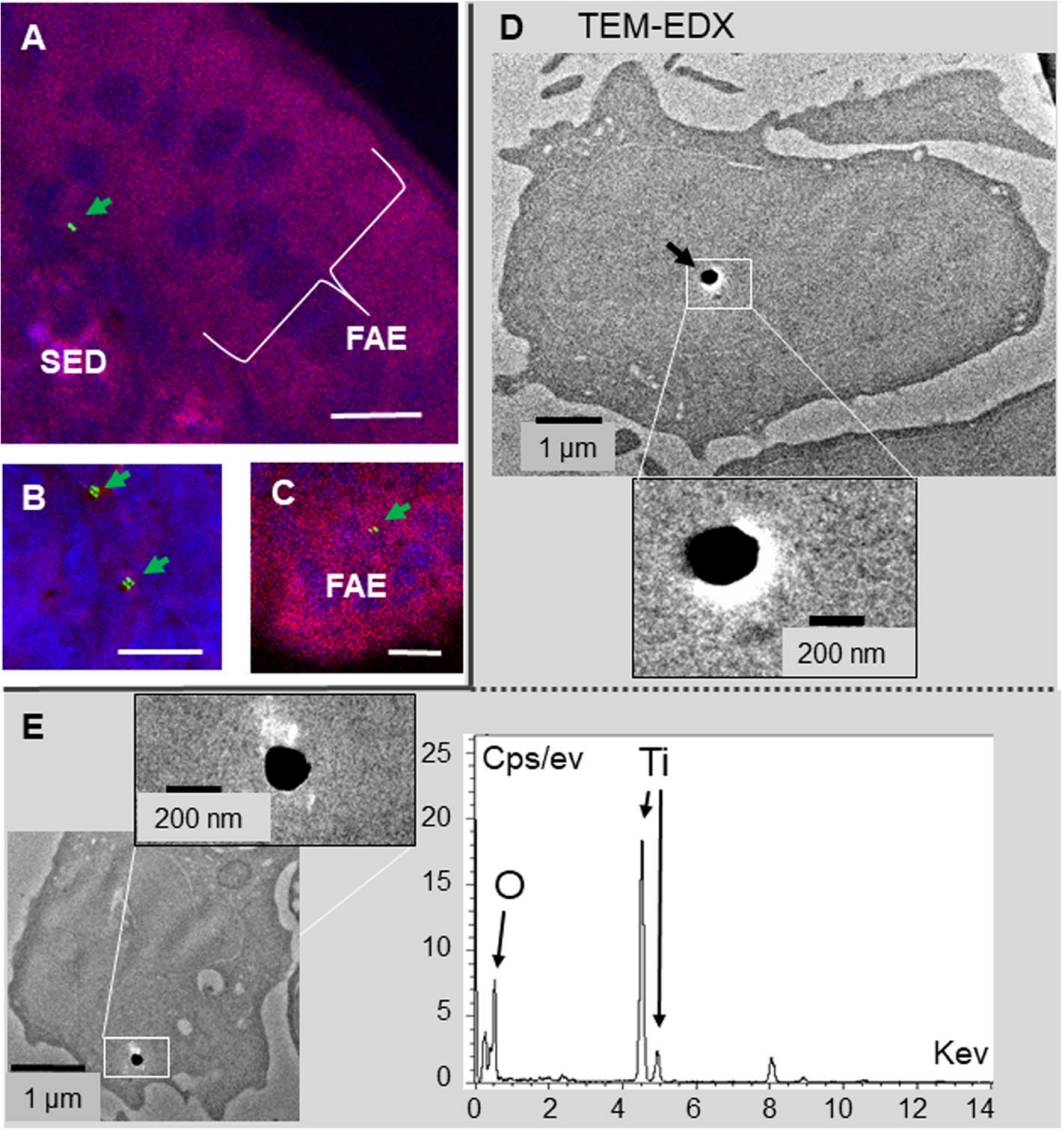
Confocal and TEM localization of TiO_2_ particles in jejunal PP, 4 h after E171 oral administration. **a-c** Confocal imaging of TiO_2_ particles (green) **(a)** in the SED, **(b)** in a lymphoid follicle (FAE) or **(c)** in the FAE monolayer**.** The tissue auto-fluorescence in shown in red and the DAPI-labelled nucleus in blue. **d, e** TEM associated to EDX analysis. TiO_2_ particles are detected as endocytosed by two separate immune cells of the PP. Bars = 10 μm in confocal microscopy, and 1 μm or 200 nm in TEM

**Fig. 4:**
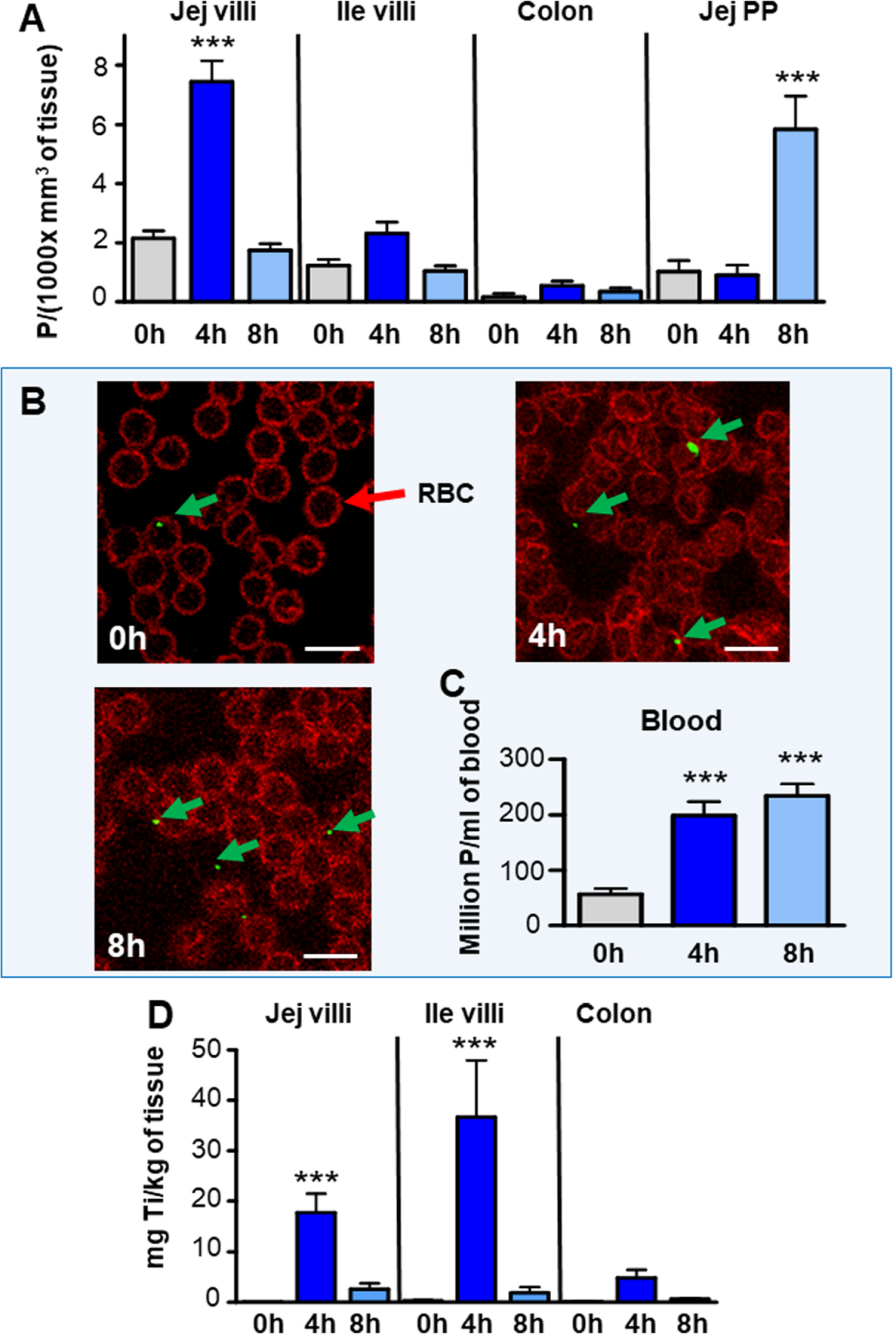
Quantification of the TiO_2_ absorption in vivo in the mouse intestine and blood at 0 h (control animals fed with water), or at 2 h, 4 h or 8 h after gavage. Number of detected particles counted: **a** in jejunal and ileal villi, in colon crypts and jejunal PP at 0 h (ctrl), 4 and 8 h after feeding; being expressed in Particles (P)/(1000xmm^3^ of tissue). Significant difference *** (*p* < 0.001) between values at 0 h versus 4 h in jejunal villi and 0 h versus 8 h in the PP (**b, c)** TiO_2_ detection in blood. **b** Confocal detection of TiO_2_ in green in blood smears recovered at 0 h, 4 h and 8 h after feeding. TiO_2_ are visible in green (as indicated with green arrows) and RBC in red. **c** Particle quantification expressed in millions of P/ml of blood; ***: significant increase for the times 4 h and 8 h as compared to 0 h (*p* < 0.001). **d** Quantification by ICP-MS of titanium contents in the jejunum, ileum and colon after in vivo absorption at 0 h, 4 h and 8 h after TiO2 oral administration, expressed in mg of titanium/kg of tissue. ***: significant increase as compared to 0 h (*p* < 0.001)

We then used confocal microscopy to assess the fraction of TiO_2_ particles absorbed by the intestinal mucosae. In control mice, a very small number of laser-reflecting particles of undefined nature were identified in all gut regions explored. In mice orally dosed with E171, an increase in the reflective particle content was observed in the jejunal and ileal villi (Fig. 2a-e), PP (Fig. 3a-c) and colon crypts (Fig. 2f). The overall particle content in jejunal villi increased from 2 h after gavage (not shown), peaked at 4 h, and returned to basal values at 8 h (Fig. 4a). At 4 h after E171 administration, the TiO_2_ particle density in the jejunal mucosa increased by 3.4-fold (*p* < 0.001) over that of control mice (Fig. 4a). A lower and non-significant trend of increased particle content was also observed at 4 h in the ileum and colon (Fig. 4a). Values decreased again close to control levels at the time 8 h in all three intestinal sections. In the jejunum, the reflective TiO_2_ particle spots displayed a mean diameter of 700 ± 59 nm (*n* = 70) and were mostly observed in the lamina propria (Fig. 2b) and in goblet cells (GCs) distributed in the epithelium (Fig. 2c), with some of them also found in enterocytes lining the gut lumen (Fig. 2d). Analysis by transmission electron microscopy energy-dispersive X-ray spectroscopy (TEM-EDX) further evidenced the absorption of TiO_2_ by revealing the presence of both Ti and O in particles detected in jejunal GCs (Fig. 2g) and enterocytes (Fig. 2h), appearing as primary particles or aggregates with respective sizes of 450 and 170 nm.

Previous studies reported increased intestinal permeability following oral treatment with pure nanosized TiO_2_ [14–16]. To assess the results of E171 feeding in this respect, we used Ussing chambers to measure the transepithelial resistance (TER) and epithelial TJ (i.e., paracellular) passage of fluorescein sodium salt (FSS) in jejunum segments recovered 4 h after E171 gavage compared to those of controls (Fig. 2i). No increase in FSS permeability or decrease in TER was observed in the jejunum of E171-treated compared to control mice, showing that the integrity of the epithelial barrier was not disrupted in our experimental conditions.

In PP, the concentration of laser-reflecting particles did not increase 4 h after E171 treatment in comparison to baseline, but reached a significantly altered level at 8 h, at which time the particle density had increased by 5.4-fold over that of controls (*p* < 0.001) (Fig. 4a). A similar profile for particle uptake was detected in more distal PP located along the ileum, but was weaker than that of the proximal (jejunal) PP (not shown). In PP, TiO_2_ particles were detected mainly in the subepithelial dome (SED) (Fig. 3a), less in the inner lymphoid tissue (Fig. 3b), and scarcely at all in the follicle-associated epithelium (FAE) containing microfold (M) cells (Fig. 3c). The size of particles in the PP (416 ± 29 nm, *n* = 71) was significantly smaller than in the blood (774 ± 45 nm, *p* < 005), the initial sonicated bolus suspension (766 ± 27 nm) and the jejunal lumen (871 ± 40 nm) (*P* < 0.001). Two TiO_2_ particles located in immune cells of jejunal PP were detected by TEM-EDX and chemically identified, measuring 210 (Fig. 3d) and 170 nm in diameter (Fig. 3e).

We also addressed the passage of TiO_2_ into systemic circulation by analyzing blood smears collected at 4 or 8 h after E171 administration or, in the case of control mice, vehicle administration. In the latter (T = 0 h), laser-reflecting particles were detected at baseline, but were significantly increased by 3.5- and 4.1-fold at 4 and 8 h after E171 administration (*p* < 0.001 vs. control) (Fig. 4b, c). At 8 h, the TiO_2_ particle density in the bloodstream was estimated to be above two hundred million particles per ml of blood.

Finally, titanium contents were analyzed by Inductively Coupled Plasma Mass Spectrometry *(*ICP*-*MS) in the blood and intestine (Fig. 4d). Despite the important detection of TiO_2_ particles by microscopy in the blood, levels remained under detection limit (< 0.02 mg Ti/kg) in samples from controls or mice sacrified at 4 or 8 h after feeding. By contrast, significant amonts were detected in the jejunum, ileum and colon at the 0 h, 4 h or 8 h conditions. Similar profiles were obtained concerning E171 absorption measured by ICP-MS or by confocal observation in the jejunum and colon with a peak at 4 h, then a substantial decrease at 8 h and higher contents in the jejunum (X6.5) compared to colon at the time 4 h. In the ileum, a similar peak was observed at 4 h, but which is twice as high as in jejunum, contrasting to confocal quantification. By extrapolation from the weight of mouse tissues, it can be estimated that approximately 0.007% of the titanium administered was present in the entire intestine at the time 4 h, which seems consistent compared to the estimated total absorption of 0.1 to 0.05%.

### Effect of lipid feeding on TiO_2_ particle uptake in vivo

**Fig. 5:**
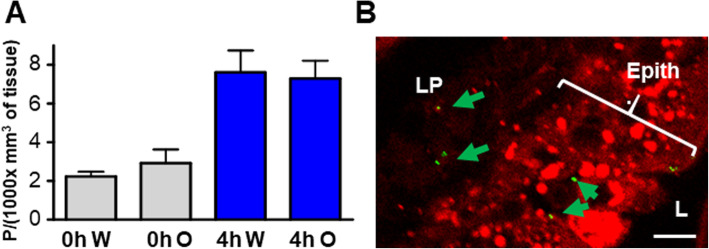
Effect of diet lipids on TiO_2_ absorption in vivo. **a** Quantification of TiO_2_ absorption in jejunal villi recovered 4 h after forced-feeding with 40 mg/ml of TiO_2_ in aqueous medium (W for Water) or in oil emulsion (O), in villi from control mouse fed with water (0 h) and **(b)** confocal identification of the distribution of TiO_2_ (green) at 4 h in the villi of oil fed mouse, the absorbed lipids (chylomicrons or lipid droplets) are stained in red by LD540 and are abundant in the epithelial monolayer (Epith) situated between the lamina propria (LP) and the lumen (L). Bars = 10 μm

Because the food additive E171 is mainly composed of TiO_2_ anatase crystal form [6, 7] with hydrophobic properties, we wondered whether the presence of lipids in the gavage bolus (i.e., to mimic a fat-rich diet) could increase the intestinal absorption of TiO_2_. To test this hypothesis, we sonicated E171 powder in water supplemented with 30% corn oil and emulsified by vigorous vortexing just before oral administration. In the jejunum samples recovered 4 h later, a time point corresponding to maximal lipid absorption [17], the particle content in the gut did not appear to differ between the aqueous and lipid vehicles (Fig. 5a). In addition, confocal imaging associated with neutral lipid staining by LD540 did not show any colocalization of intestinal lipid droplets and TiO_2_ particles (Fig. 5b), suggesting different routes of absorption.

### Ex vivo analysis of particle translocation pathways

**Fig. 6:**
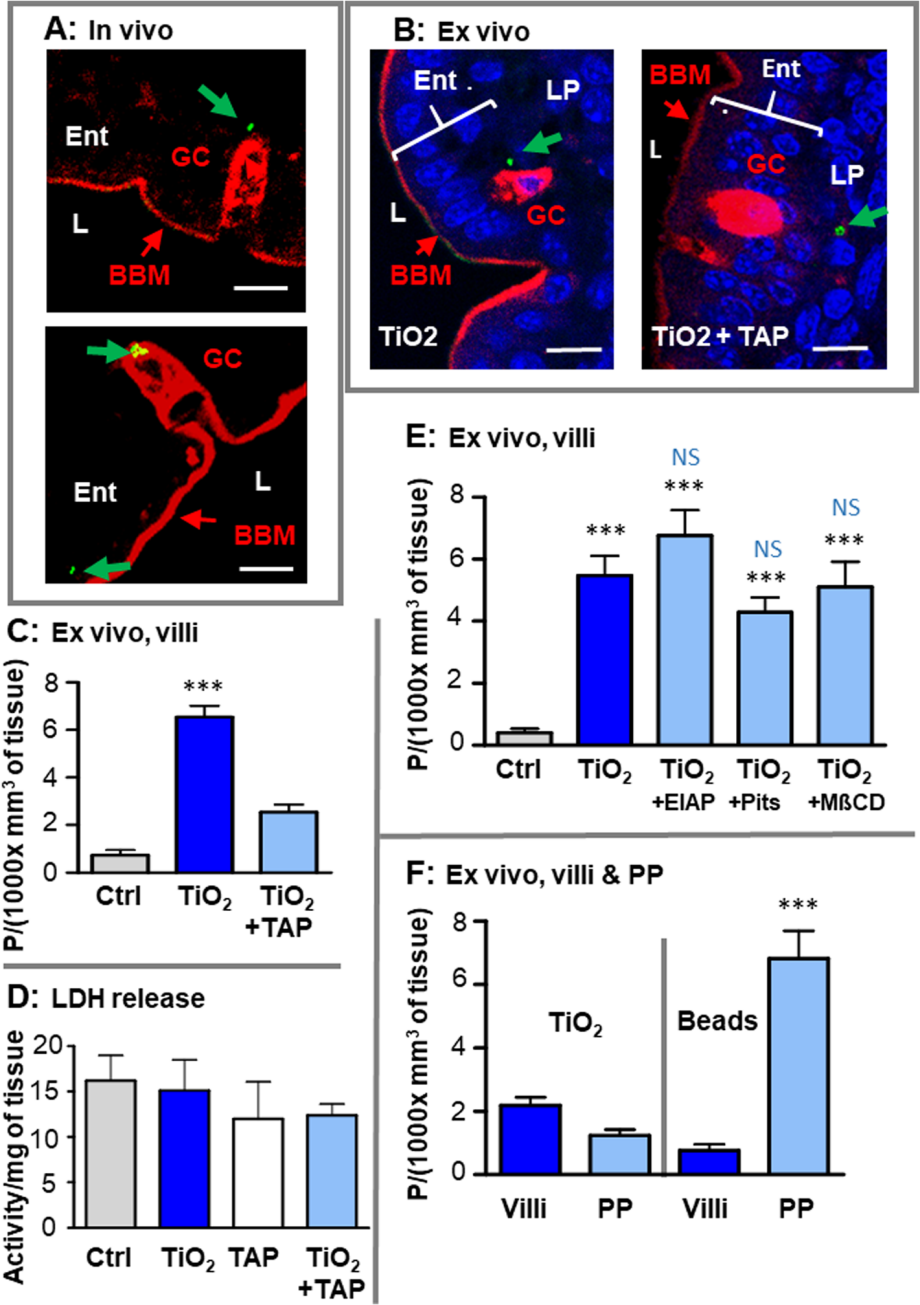
Confocal detection of TiO_2_ and of epithelium surfaces in the jejunum after in vivo or ex vivo exposure to E171, and comparative analysis of ex vivo absorption of TiO_2_ particles and of polystyrene beads. **a, b:** after TiO_2_ exposure, in vivo (4 h) **(a)** or ex vivo (30 min) **(b)**, GC and apical surface of epithelial cells were labelled in red with the FM-1-43-FX dye; TiO_2_ is visible in green (arrows) and the nuclei stained with DAPI (blue). BBM = Brush border membrane of enterocytes (Ent), L = lumen, LP = lamina propria. **(c, d, e):** Jejunal loops were incubated ex vivo without (Ctrl) or with TiO_2_ (E171 300 μg/ml), alone or in the presence of the TJ blocker TAP **(c)** or the endocytosis blockers EIAP, Pitstop or MβCD **(e)**. Results were expressed in number of particles (P)/(1000 x mm^3^ of tissue). After incubation the luminal medium was recovered to measure the release of LDH from tissues, and was expressed in activity/mg of tissue **(d)**. In **c** ***: significantly different for TiO_2_ alone versus Ctrl or TiO_2_ + TAP (*p* < 0.001). In **e**) ***: significantly different from Crtl (*p* < 0.001), and NS: not significant versus TiO_2_ alone. **f** Quantification of ex vivo absorption of TiO_2_ or polystyrene beads in jejunal villi and PP; ***: significantly different between villi and PP (*p* < 0.001)

We analyzed the transepithelial passage of TiO_2_ in the proximal gut using ligated intestinal loops exposed to E171 in anesthetized mice (Fig. 6), with and without specific blockade of paracellular or transcellular transport [18–21]. The quantification of laser-reflecting TiO_2_ particles was focused on jejunal villi, in which we observed maximal particle absorption in vivo with our confocal technique. In the presence of TiO_2_, the particle uptake was blocked by 66% in jejunum in the presence of tissues treated with 100 mM (2,4,6-triaminopyrimidine) TAP compared to control tissues (2.202 ± 0.295 vs. 6.540 ± 0.475 particles/(1000 x mm^3^ of tissues), respectively; *p* < 0.001) (Fig. 6c). The compound TAP acts on tight junctions (TJs) between enterocytes, limiting their permeability to macromolecules in addition to water and small inorganic ions [20, 21]. The effect of TAP on TiO_2_ absorption indicates a major and rapid contribution of the paracellular TJ pathway to transepithelial TiO_2_ uptake during the 30 min of incubation. To assess potential cell lysis during ex vivo incubations, we measured the release of cytosolic lactate dehydrogenase to the resulting luminal milieu from incubations without or with the presence of TAP, TiO_2_ particles and TiO_2_ + TAP. Similar amount of LDH release was observed in all conditions as expressed in LDH activity/mg of tissue, showing no toxicity of TiO_2_ and TAP (Fig. 6d). When endocytosis inhibitors were applied, non-significant trend-level decreases in macropinocytosis, clathrin-dependent endocytosis, and raft-dependent endocytosis were revealed (Fig. 6e), suggesting a minor contribution of transcellular transport to the movement of TiO_2_ particles across the epithelium over the same exposure duration with E171.

We then compared the absorption rates of laser-reflective TiO_2_ particles and carboxyl-polystyrene (CP) beads on jejunal loops (Fig. 6f). Amorphous beads are mainly absorbed by endocytosis through M cells of the PP and far less in intestinal villi [22, 23], as confirmed herein, showing maximal bead absorption at PP sites in the jejunum rather than villi. In clear contrast, the absorption rate of TiO_2_ particles was similar between jejunal PP and villi, and remained low compared to CP beads regardless of the absorption site.

### Imaging of TiO_2_-particle-permeable zones on intestinal surfaces

In this study, jejunal segments collected 4 h after E171 oral administration or ligated jejunum loops exposed to E171 were filled with the non-membrane-permeable dye FM-1-43FX (FM) to stain the apical epithelial surface [24]. This was performed at a temperature of 0 °C, at which endocytosis is completely inhibited, to label surfaces that are in direct contact with the intestinal lumen. No FM entry was detected between enterocytes following E171 treatment, attesting to the integrity of the epithelial TJ barrier as herein reported. In contrast, we consistently observed passage of the FM dye around GC plasma membranes, both in vivo and ex vivo, suggesting the existence of additional paracellular passage of the dye. This passage appeared to be independent of TJ function, since it was observed with and without the TJ blocker TAP. We then imaged the GCs in the E171-treated mice or jejunal loops. The presence of TiO_2_ particles in these GC passages has frequently been observed in both the presence and the absence of TAP, suggesting an additional, TJ-independent paracellular pathway allowing lumen-to-mucosa TiO_2_ uptake in addition to enterocyte TJ permeability.

Finally, previous studies showed that GCs also transport luminal antigens by a specific pathway known as GC-associated antigen passages (GAPs), in which antigens are endocytosed by the GC then delivered directly to underlying dendritic cells [25, 26]. Herein, we observed TiO_2_ particles inside GCs by TEM analysis (Fig. 2g), supporting the concomitant absorption of TiO_2_ particles through endocytosis by GAPs and TJ-independent paracellular GC permeability.

## Discussion

Food-grade TiO_2_ is authorized in the EU (E171) *ad quantum satis*, resulting in an estimated TiO_2_ content of 0.2 to 4 mg/kg of body weight (BW)/day in the human diet, reaching 32 mg/kg BW/day in the 95th percentile of children [3]. Although most toxicokinetic studies concluded that the intestinal absorption of TiO_2_ was low, i.e., 0.1–0.6% of the administered dose [8–11], it is presumed that the corresponding uptake of TiO_2_-NPs by number is high, due to the high prevalence of NPs in currently available batches of E171 (10–45% of particles) [1, 5–7, 27] in their primary state. In humans, TiO_2_ particles have been found postmortem in the liver and spleen, with at least 25% in the nanosize range, and most of them were assumed to originate from intestinal exposure [28]. In the current study conducted in mice, the high laser reflectance index of TiO_2_ was used to easily detect and follow its uptake in the mouse intestine, thanks to confocal microscopy permitting particle detection in tissues, as well as in physiological fluids. In the gut, given the low density of TiO_2_ particles detected in the mucosa after E171 oral treatment [7], observations over large tissue areas are required for an accurate evaluation of particle uptake. This was achieved using low magnification under confocal microscopy and by setting the detection limit at 200 nm/pixel, excluding the detection of nanosized particles. A higher resolution of 50 nm/pixel was used on a case-by-case basis to refine particle size measurement and for nanometric particle detection (i.e., < 100 nm) as was also done by TEM. In this study, the increase in the number of particles detected by confocal imaging after the oral administration of E171 in mice was assumed to correspond to the absorption of TiO_2_, since no other source of particles was given to the mice, and the laser-reflecting particulate matter was abundant in the intestinal lumen after oral treatment, but absent in the water-fed controls. The absorption of TiO_2_ was further demonstrated by the measurement of titanium content in the intestine by ICP-MS, after oral administration in vivo. Moreover, lumen-to-blood passage of the particles was observed in this study, with kinetics close to the absorption of a food-grade TiO_2_ formulation ingested by human volunteers [13]. Our results in mice thus substantiate these data, showing predominant uptake in the small bowel and suggesting specific routes for particle absorption. Although the amount of TiO_2_ herein administered to mice was 40 mg/kg BW, exceeding the average daily human intake 4- to 10-fold [1, 3], this was necessary to achieve statistical significance for uptake quantification by confocal analysis. In addition, the TiO_2_ particles were administered in water by gavage in mice fed a normal diet, which left the particles free and available to the gut epithelium. This exposure scenario is credible for humans when TiO_2_ is used as a coating for food products (e.g., candies, chewing gums) or in liquid matrices (ice and sauces), allowing the rapid suspension of (nano) particles in the saliva before their ingestion [1, 2]. It has been suggested, based on the exposure of TiO_2_ in vitro to reconstituted gastric and intestinal juices, that the low pH in the gastric milieu or biliary salts and proteins in the intestinal lumen may impact the size of TiO_2_ particulate matter, leading to substantial reagglomeration in the gut lumen to large particle clusters with sizes that could limit their absorption [14]. In contrast, we report similar sizes between the laser-reflective TiO_2_ particles recovered from the lumen of the upper gut and those observed in the gavage bolus, indicating no further agglomeration of TiO_2_ particles during intestinal transit as previously reported in rats [7]. Second, smaller sizes were reported in the colonic lumen than in the small intestine; this suggests a decrease in the agglomeration state of TiO_2_ from the proximal part to the more distal part of the intestine. Interestingly, we also show that the presence of lipids in the mouse bolus and their absorption by the upper intestine did not interfere with the bioavailability of TiO_2_; there was an independent distribution of lipids and E171 particles in the tissue. We also checked whether oral E171 feeding in mice might have a deleterious impact on gut epithelial integrity that would artificially increase and bias their absorption level, possibly due to the abrasive potential of TiO_2_ particles. In the current study, no change in intestinal permeability was observed at the time of maximal TiO_2_ uptake (i.e., 4 h after gavage), showing that our treatment conditions were safe for gut integrity, as shown in rats [7]. Similar, no epithelial damage was detected in the presence of TiO_2_ by confocal imaging associated with FM surface labeling in vivo or ex vivo.

In the current study, after a single oral dose, the TiO_2_ particle absorption appeared preponderant in the small intestine, being detected mostly in the jejunum, then in the ileum and poor in the colon as measured by confocal microscopy, and preponderantly in the ileum, less in the jejunum and low in colon by ICP-MS analysis. Absorption in the small intestine was transient, with particle number in the villus mucosa reaching a maximum 4 h after oral intake before returning to low level at 8 h. This suggests that the majority of TiO_2_ particles are absorbed without accumulation in the gut mucosa following a single administration, in contrast to the persistent particles detected in the intestinal wall after 1 week of repeated doses [7]. In our study, the kinetics of particle translocation was confirmed by a 3- and 4-fold increase in the number of particles recovered in blood at 4 and 8 h after dosing, respectively. This is in full agreement with data in humans, where food-grade TiO_2_ particles were detected in the bloodstream in increasing amounts for up to 6 h after ingestion and gradually decreased at 8 and 10 h [13]. The presence of TiO_2_ in blood could not be detected by ICP-MS in our experimental conditions, possibly because of the small amounts present in mice. The preponderant absorption of TiO_2_ in the ileum, compared to the jejunum was established by ICP-MS, but not detected by confocal microscopy where the major uptake was detected in the jejunum. Titanium contents at 4 h are much higher than the control levels (0 h) for jejunum (× 120), ileum (× 250) and colon (× 40), which are larger increases than those found by confocal microscopy. This could be explained by the fact that confocal techniques have failed to detect small particles in all tissues, but especially in the ileum where they seem particularly present. In the end, the jejunum absorbs about half as much TiO_2_ as the ileum, but it is twice as long and heavy, balancing the respective amounts of E171 finally absorbed by the jejunum or ileum as a whole.

In the current report, we also compared the translocation rate of particles between jejunal villi (i.e., a regular epithelium composed of enterocytes) and PP, which are involved in gut immunity. Microfold cells (M cells) lining the PP dome are specialized epithelial cells for transcytosis of antigens across the follicle-associated epithelium (FAE), leading to downstream organization of the immune response. In contrast to jejunal villi, TiO_2_ uptake was not found in the PP at 4 h but was detected at 8 h, mainly in the jejunal PP which are more aboundant in mice than ileal PP. Moreover, although M cells are thought to be common absorption sites for luminal compounds of various sizes, including inorganic particles [25, 26, 29–32], our study showed that the local uptake capacity of TiO_2_ particles per unit surface area of PP was similar to that observed in the regular intestinal villi. In addition, using intestinal loops, we also provided evidence of a very low absorption rate of TiO_2_ particles in the PP when compared to the uptake capacity of polystyrene beads of a similar size range. FAE was capable of transporting polystyrene beads at a rate up to 10-fold higher than intestinal villus uptake, while the PP absorption rate of TiO_2_ particles remained significantly lower and was not different from villi. Finally, the number of PP in the small intestine is low, which limits the total surface area of FAEs in contact with lumen, here estimated at about 1/100 of the total area of the small intestine. It can therefore be concluded that TiO_2_ is overwhelmingly absorbed by the small intestinal villi. The total amount of TiO_2_ absorbed in the colon after this single force-feeding is much more modest at 4 h and not significant in both confocal or ICP-MS analysis. Although it may be slightly delayed in time compared to the small intestine, it is further reduced to low levels at 8 h after administration.

Using confocal imaging of large tissue fields, it is not possible to evaluate the exact size of primary particles translocated into tissues (and blood). This was possible, however, at high magnification, when particle size measurement was performed simultaneously by laser reflectance and conventional light analysis of opaque TiO_2_ crystals. We thus determined that most TiO_2_ material found in the gut mucosa correspond to aggregates of particles and agglomerates ranging in size from 200 to 2300 nm in jejunal villi and 200 to 1200 nm in PP. It can be assumed that TiO_2_ particles can translocate directly as micrometric and submicron-scale agglomerated forms from the gut lumen, while we cannot exclude the hypothesis of a lumen-to-mucosa passage of isolated nanoparticles that also reagglomerate once penetrated into tissues, as reported in vitro [14, 33–40]. However, most particles were detected without apparent clustering, but exhibited a fairly good dispersability relative to each other, and similar sizes in the jejunum lumen, villi and blood, suggesting their uptake without significant agglomeration in the tissues. Aggregation is possible anyway, but hard to establish.

Furthermore, we used specific pharmacological inhibitors of paracellular and transcellular (endocytosis) permeability pathways to study the route(s) involved in the lumen-to-mucosa transport of TiO_2_ particles in jejunal villi. Experiments conducted ex vivo on intestinal loops in the presence and absence of the TJ blocker TAP revealed significant inhibition of TiO_2_ absorption (i.e., by 66% after 30 min of incubation), demonstrating that the paracellular route is a major pathway governing transepithelial TiO_2_ passage. This could explain why most of the particles were found in the lamina propria beneath the epithelium, both in vivo and ex vivo, without apparent sequestration into epithelial cells. The multiprotein TJ complex forms a selective permeable seal between adjacent epithelial cells by linking the apical and basolateral membrane domains of enterocytes [30, 41]. In healthy conditions, TJs maintain intestinal barrier function (IBF) against deleterious antigens while regulating the permeability of ions, nutrients, and water. Altogether, these data demonstrated that the transepithelial transport of TiO_2_ particles in the jejunal region mainly occurs through intercellular TJs. This is consistent with our previous study in E171-treated rats identifying TiO_2_-NPs by TEM in the space between enterocytes after 1 week of oral treatment [7]. Finally, regarding the data obtained with other blockers of transcellular pathways, either macropinocytosis, clathrin-dependent endocytosis or raft-dependent endocytosis, demonstrated that none of these inhibitors displayed a significant impact on TiO_2_ uptake over the 30 min of ex vivo experiments. This indicates that the transcellular uptake of TiO_2_ across the intestinal epithelium is too weak to achieve statistical significance during this short period of treatment, in contrast to paracellular passage. However, in in vivo studies, substantial endocytosis was observed using TEM analysis, involving not only GCs or villus enterocytes, but also immune cells within the PP, which is in support of a delayed accumulation of TiO_2_ in the PP. Endocytosis within the tissue can also increase the retention of particles or even promote agglomeration inside endosomes [7, 16–18, 42], and could explain part of the basal level of intra-intestinal particles recovered in the control group, which did not receive TiO_2_.

Finally, approximately one-third of TiO_2_ particles were still shown to translocate in the jejunum after TAP treatment, suggesting other (i.e., TJ-independent) route(s) for rapid particle uptake. Translocation by enterocyte persorption could be involved in TAP-resistant uptake of TiO_2_, as shown with gold nanoparticles [43]. In our study, although persorption sites were experimentally identified, which were underlined by labeling with the non-permeable fluorescent dye FM-1-43FX, while no TiO_2_ translocation was noticed in the vicinity of these sites (not shown). On the other hand, we reported passage of the FM dye close to mucus-producing GCs, ex vivo and in vivo*,* in the presence and absence of TAP, as well as when endocytosis was blocked at 0 °C. This first result showed passive diffusion of the dye by a paracellular route independent of TJ function. Interestingly, our results show TiO_2_ particles close to the GC basolateral membranes whether the inhibitor TAP is present or not. It is reasonable to propose this transepithelial GC-related pathway is a complementary and TJ-independent route for TiO_2_ particle absorption. It is also suggested that the absence of TJ seals could allow the uptake of large particles, which could partly explain the translocation of TiO_2_ agglomerates of larger sizes in the villi than in the PP.

## Conclusion

Food-grade TiO_2_ particles are mainly absorbed in the small intestine after a single oral administration, where they cross the regular epithelium of the small bowel villi, rather than the PP, as their main translocation site. Ex vivo, using intestinal loops, we have demonstrated that the absorption of food-grade TiO_2_ through the epithelial barrier occurs mainly in a paracellular pathway between enterocytes or around the GCs. Most of the particles recovered in vivo or ex vivo from within tissues are found in the lamina propria of the villi and in the subepithelial domes of the PP, without substantial epithelial transcytosis, a pattern that is also consistent with paracellular transport as the major entry route. Finally, because the size distribution of TiO_2_ particulate matter detected by confocal imaging did not show significant differences among the initial oral bolus, the jejunal lumen and villi, and the blood, TiO_2_ particle uptake by the intestine appears to be indiscriminative with respect to size. The absorption measured in this study represents the absorption of free TiO_2_ particles, without a food bolus, which can increase it. However, it should be noted that using ICP-MS, at time 4 h, an absorption close to 0.007% of the administered amount was measured. This rate remains in the same order of magnitude as the 0.1 to 0.05% described as being absorbed overall after oral administration. This still represents about tens of mg of Titanium/kg of intestine, under our experimental conditions (ei 40 mg/kgBW in mice).

## Methods

### Particle preparation

The E171 sample was obtained from a French commercial supplier of food coloring. The TiO_2_ particle suspensions (> 95% anatase, [6]) were prepared in milliQ water following the generic Nanogenotox dispersion protocol [44], and the physicochemical properties of the particles in the E171 batch have been characterized in previous studies [6, 7, 45].

### Animals and experimental design

Adult C57BL/6 mice (12–18 weeks) were purchased from Janvier Labs (France). All animal experiments were performed in accordance with the guidelines of European legislation (Council Directive 2010/63/UE) and French Decree 2013–118 on the protection of animals used for scientific purposes, and were approved by the Local Animal Care and Use Committee (TOXCOM-0036-EH-EH) of Toulouse Midi-Pyrénées (agreement CEEA-86). The animal facilities used are licensed by the relevant local authorities for rodents (agreement C31 555 13). In the first series of experiments, mice (*n* = 4 mice/group) received a single dose (200 μl) of either E171 at 40 mg/kg of body weight (BW) or 200 μl of vehicle (water) by intragastric gavage. At different times after gavage (2, 4, 8, and 24 h), the animals were sacrificed, and the intestine (jejunum, ileum and colon) was recovered. To obtain jejunal or colonic intraluminal contents, we collected a segment of tissue and opened it longitudinally. The intraluminal substance, present just above the mucosa, was recovered by gently scraping, then spread on a glass slide and air dried. The remaining intestinal sections were washed in 0.9% NaCl and fixed in 4% formaldehyde, equilibrated in 30% sucrose in phosphate-buffered saline, frozen in liquid nitrogen and stored at − 80 °C. Blood smears were also prepared and air dried at the same time points. In several experiments, the gavage solution from sonicated E171 particles was equilibrated in 30% corm oil, and vigorously vortex for emulsification just before oral delivery. This was performed to analyze if luminal lipids, being themselves absorbed in the jejunum, could influence overall TiO_2_ absorption. This was studied because we experimentally observed that lipids, in emulsions, attract E171.

### Measurement of titanium concentration in tissues and blood by ICP-MS

Inductively coupled plasma mass spectrometry (ICP-MS) analysis was performed of blood samples (0.3 to 0.7 ml), 10 cm fragments of jejunum, ileum, and colon from mice treated by oral feeding with H_2_O (0 h) or TiO_2_ suspension at 40 mg/kg of BW, and sacrificed 4 or 8 h later (*n* = 8 for each condition). For total titanium determination, samples (E171 suspensions or whole organs) were placed in conical tubes (Greiner) with 0.375 ml HCl (Optima, Fisher), 0.875 ml HNO3 (Optima, Fisher) and 0.05 ml of HF (ultrapur, Merck). The samples were digested in a heating block (Digiprep, SCP SCIENCE) during 180 min at a temperature of 105 °C. Total titanium concentrations were measured with a high-resolution ICP-MS spectrometer (Element II, Thermo Scientific) equipped with a baffled cyclonic spray chamber and a conical nebulizer. Analytical masses were 47Ti in medium resolution mode and 103Rh (as internal standard). The system was tuned on a daily basis to ensure maximum titanium sensitivity and quantitative determinations were done by external calibration.

### Intestinal loop experiment

In a second series of experiments, the mice were anesthetized by intraperitoneal injection of a mixture of xylazine at 0.014%/kg of BW and ketamine 72 mg/kg of BW), and body temperature was maintained during surgery at 36–38 °C using a heating pad. A small abdominal incision was made to expose the small intestine, and closed midjejunal loops (10 cm in length) were isolated by the addition of sutured catheters at each end, permitting changes to luminal media or closure of the loops during incubation. The loops were filled with either PBS alone (control) or PBS containing one of the following inhibitors (from Sigma-Aldrich, Saint-Quentin-Fallavier, France): TAP at a final concentration of 100 mM to block tight junctions, EIPA (5-(N-ethyl-N-isopropyl) amiloride) at a final concentration of 100 μM to inhibit macropinocytosis, Pitstop 2 at 30 μM to block clathrin-mediated endocytosis, and MβCD (methyl-β-cyclodextrin) at 17 μM to inhibit raft-dependent endocytosis. Thirty minutes later, the lumen contents were rinsed away and replaced with the same PBS solution with or without 300 μg/ml of sonicated E171 TiO_2_ for a further incubation period of 30 min. We analyzed in parallel the cell lysis by measuring the cytosolic release of lactate dehydrogenase (LDH; as described in [46], using sodium pyruvate, NADH and spectrophotometry) in the lumen recovered after ex vivo experiments performed in control milieu, in the presence of TiO_2_, TAP or both of them. Results were expressed in LDH activity/sec/mg of protein in the corresponding tissue.

The absorption of 300 μg/ml TiO_2_ and 2 μg/ml fluorescent polystyrene beads (400–600 nm diameter, Spherotech, Lake Forest, IL, USA) was also compared after incubation for 30 min in jejunal loops. For each condition, the experiment was performed in 4 mice spread over 3 independent experiments. Finally, the intestinal loops were recovered, washed and fixed as described above.

### Confocal microscopy and micro-X-ray fluorescence imaging

Tissue samples were cryosectioned (25 μm thickness), air dried, fixed in acetone at − 20 °C (5 min), rehydrated in PBS (10 min), and then mounted in ProLong Gold antifade medium with DAPI (4′,6-diamidino-2-phenylindole, Life Technologies, France). Tissue sections were examined under a confocal microscope (Leica SP8, Nanterre, France) at 488/BP 488–494 nm to detect light reflection by TiO_2_ particles and at 514/BP 560–660 nm to monitor autofluorescence in the tissue. To ensure that the particles detected were truly in the intestinal section and were not exogenous contaminants introduced during tissue sectioning and rehydration, we took into account only the particles present in the central 8 μm of the 25-μm depth of the entire section. Particle number per volume (surface x depth) was counted and expressed in multiple of mm^3^. Similar measurements of TiO_2_ light reflection at 488/BP 488–494 nm were performed to determine the sizes of the particles present in the E171 water suspensions prepared for gavage to the mice, in the luminal contents (jejunum and colon) and in the blood. All solutions were spread on glass slides, dried and mounted in ProLong medium. Concerning blood smears, the numbers of particles and of red blood cells (RBCs) were counted for each microscope field. The volume of blood corresponding to the analysis was then calculated from the number of RBCs given that mouse blood contains 10 × 10^9^ RBCs/ml. The particles were detected using a 40 or 63× objective and a magnification factor leading to a pixel width ranging from 200 nm (to screen large tissue areas) to 50 nm (to determine particle size). The size measurement of the TiO_2_ particles at this high magnification allowed dual determination by confocal laser reflection and then by classic bright-field observation, with similar results.

We performed in several fresh jejunal fragments, recovered at sacrifice after in vivo or ex vivo experiments, an incubation with the non-permeable fluorescent dye FM-1-43FX (Fisher Scientific™, Toulouse, France). For that, the tissues were washed with 0.9% NaCl to remove luminal TiO_2_, were cooled to 0 °C in ice, ligatured at both extremities. They were then filled with 10 μg/ml of the FM dye in PBS, incubated for 5 min in ice as previously described (22), washed, fixed, frozen and analyzed as described above. This permitted to label any membrane in contact with the luminal milieu, since all intracellular uptake by endocytosis was blocked by incubation at 0 °C.

### Transmission electron microscopy and EDX analysis

Jejunal villi and Peyer’s patches recovered from mice 4 h after gavage were analyzed by TEM. Tissue samples measuring 1 mm^3^ were fixed in 2.5% glutaraldehyde-2.5% paraformaldehyde in 0.1 M cacodylate buffer (pH 7.4) overnight (+ 4 °C) and post-fixed in 1% osmium tetroxide solution (2 h, + 4 °C). They were dehydrated through a graded series of ethanol and embedded in Epon-Araldite resin. Ultrathin sections (80 nm) were cut (Ultracut Leica), mounted on copper grids and stained with uranyl acetate and lead citrate. Observations and nanoparticle characterization were carried out with a JEOL JEM 1400 or JEOL JEM 2100F TEM operated at 200 kV coupled to an energy-dispersive X-ray spectroscopy (EDX) analysis system.

### Measurement of intestinal permeability in Ussing chambers

Two groups of four mice received a single dose (in a volume of 200 μl) of either 40 mg/kg of TiO_2_ or water by gavage. Four hours later, the animals were sacrificed, and the two separate segments of the jejunum from each mouse were isolated and mounted in Ussing chambers (Physiological Instruments, San Diego, CA), with an aperture size of 0.125 cm^2^. Each half-chamber contained 1 mL of Krebs buffer (118 mM NaCl, 4.7 mM KCl, 1.2 mM KH_2_PO_4_, 1.2 mM MgSO_4_, 25 mM NaHCO_3_, 8.3 mM glucose, 2.5 mM CaCl_2_), continuously gassed with 95% O_2_–5% CO_2_. Ag–AgCl electrodes were used as short-circuiting electrodes and to measure the transepithelial electrical properties of the tissues throughout the experiment, including the transepithelial potential difference, short-circuit current, and transepithelial resistance (TER), permitting the assessment of tissue viability. After 20 min of equilibration, fluorescein sodium salt (FSS) was added on the mucosal side (40 μg/ml final concentration). The paracellular permeability was assessed by measuring both the TER and the fluorescence that had reached the serosal side after 60 min.

### Statistical analysis

The results are expressed as the mean ± s.e.m. Statistical significance was assessed using Prism 4 software (GraphPad) by non-parametric one-way ANOVA (Kruskal-Wallis test) followed by post hoc tests. A *P* value of < 0.05 was considered significant.

## Supplementary information

**Additional file 1: **Fig. S1: Identification **(A,B)** and size measurement **(C)** by TEM of the TiO_2_ particles and agglomerates present in the E171 sonicated suspension used for oral administration (*n* = 354).

**Additional file 2.** Fig. S2: Confocal identification of laser reflective particles present in the jejunal (A) or colonic lumen (C) at 4 h after feeding, and absent in control (ctrl) lumens (B,D). Bars = 10 μm

## Data Availability

Supplement data are avalable to upload at the end of the PDF format submitted.

## Abbreviations

BW: Body weight
DAPI: 4′,6-diamidino-2-phenylindole
EIPA: 5-(N-ethyl-N-isopropyl) amiloride
EU: European Union
FFS: Fluorescein sodium salt
FAE: Follicle-associated epithelium
GAP: GC-associated antigen passage
GC: Goblet cell
IBF: Intestinal barrier function
MβCD: Methyl-β-cyclodextrin
NP: Nanoparticle
P: Particle
PBS: Phosphate buffered saline
PP: Peyer’s patches
RBC: Red blood cell
SED: Subepithelial dome
TAP: 2,4,6-triaminopyrimidine
TEM: Transmission electronic microscopy
TEM-EDX: Transmission electron microscopy energy-dispersive X-ray spectroscopy
TER: Transepithelial resistance
TJ: Tight junction
