# Supplementary material for: Jejunal villus absorption and paracellular tight junction permeability are major routes for early intestinal uptake of food-grade TiO2 particles: an in vivo and ex vivo study in mice
Source: Part Fibre Toxicol. 2020 Jun 11;17:26. doi: 10.1186/s12989-020-00357-z (PMC7345522; doi:10.1186/s12989-020-00357-z)
Supplement: Supplementary file 1 — Additional file 1: Fig. S1: Identification (A,B) and size measurement (C) by TEM of the TiO2 particles and agglomerates present in the E171 sonicated suspension used for oral administration (n = 354). [file 12989_2020_357_MOESM1_ESM.pdf]

Figure S1

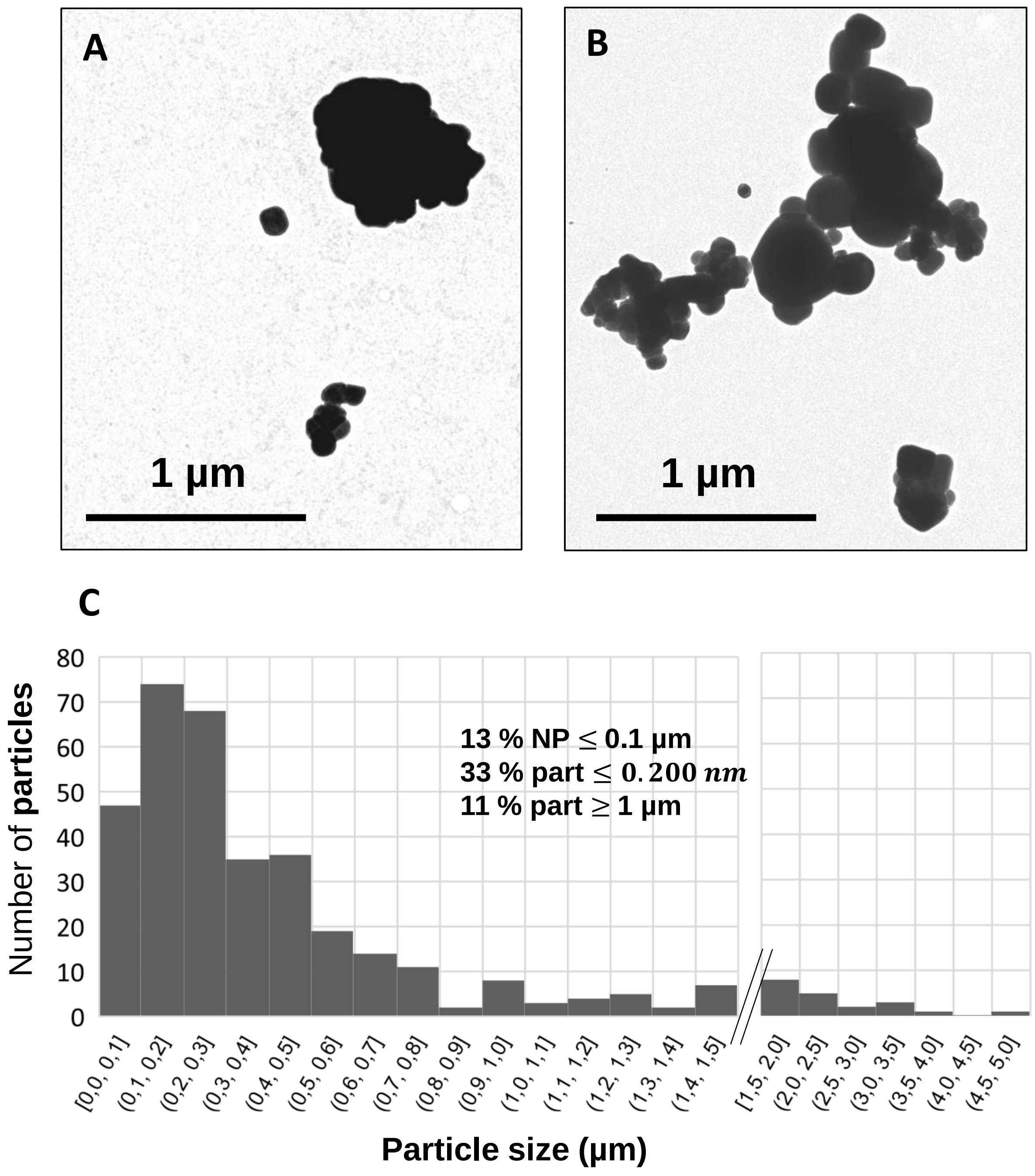

Fig S1 : Identification **(A,B)** and size measurement **(C)** by TEM of the TiO2 particles and agglomerates present in the E171 sonicated suspension used for oral administration (n=354).

Figure S2

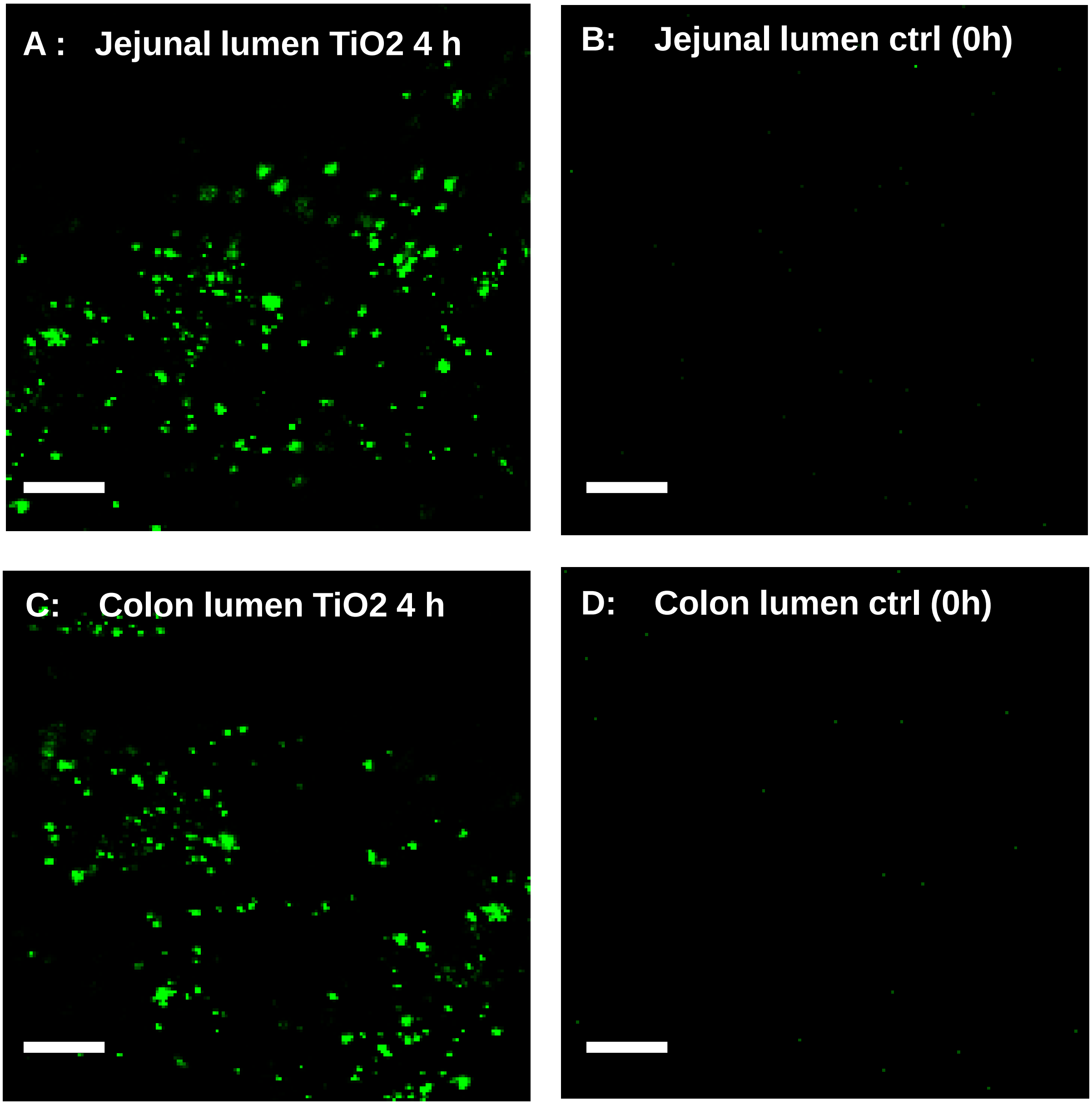

Fig S2 : Confocal identification of laser reflective particles present in the jejunal (A) or colonic lumen (C) at 4 h after feeding, and absent in control (ctrl) lumens (B,D). Bars=10 µm
