# Supplementary figures and images for: Jejunal villus absorption and paracellular tight junction permeability are major routes for early intestinal uptake of food-grade TiO2 particles: an in vivo and ex vivo study in mice
Source: Part Fibre Toxicol. 2020 Jun 11;17:26. doi: 10.1186/s12989-020-00357-z (PMC7345522; doi:10.1186/s12989-020-00357-z)

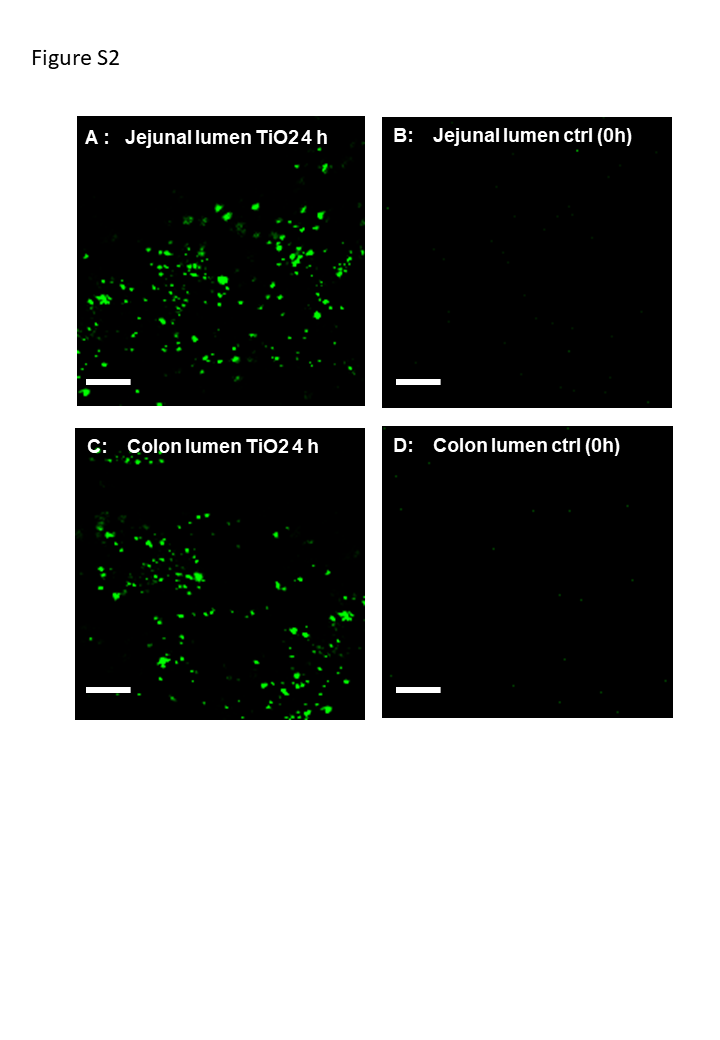

Supplement: Supplementary file 2 — Additional file 2. Fig. S2: Confocal identification of laser reflective particles present in the jejunal (A) or colonic lumen (C) at 4 h after feeding, and absent in control (ctrl) lumens (B,D). Bars = 10 μm [file 12989_2020_357_MOESM2_ESM.tif]
